# Surgical treatment for pulmonary metastases in urogenital cancers

**Published:** 2014-09-25

**Authors:** IM Radulescu, R Popescu, MM Cirstoiu, I Cordos, D Mischianu, CF Cirstoiu

**Affiliations:** * Clinic of Thoracic Surgery, "Marius Nasta" National Institute of Pneumology, Bucharest; ** Obstetrics and Gynecology Department, University Emergency Hospital, Bucharest; *** Urology Department, "Dr. Carol Davila" Central Military University Emergency Hospital, Bucharest; **** "Carol Davila" University of Medicine and Pharmacy, University Emergency Hospital, Bucharest

**Keywords:** pulmonary metastases, urogenital cancer, pulmonary resection

## Abstract

Abstract

Introduction. The malignant disease’s ability to metastasize remains one of the major obstacles when treating patients with cancer. The indication of metastasectomy is currently limited to patients undergoing treatment of the primary tumor. Resections for lung metastases of high selected patients with urogenital cancer present minimal risks and can prolong survival. Prognostic factors that determine which patients will benefit from surgery are still unclear.

Material and results. This article presents a retrospective analysis of patients who underwent lung metastases resection between 2008 and 2013 in our clinic. Among 148 patients, 8 (5.41%) had lung metastases after urologic cancers (UC), 18 (12.16%) after genital cancers (GC), 13 (8.78%) after breast tumors and 109 (73.65%) had lung metastases from other type of tumors. The overall 6 months survival was 100% for UC, 94.44 for GC, 84.62% for BC and 87.16% for others.

Discussion and conclusion. The criteria for surgery proved to have a positive predictive value and what should be considered are the following: prolonged disease-free interval (DFI), unilateral disease, the absence of systemic pathologies, oncological margins resecability and less than 3 radioimagistic detectable metastases. A negative prognosis was observed in those with primary tumor in the cervix, at least 3 metastases and a tumor larger than 3 cm. To determine how to select surgical candidates for pulmonary metastasectomy more precisely, further analysis of prognostic factors is evident and the need for a prospective, randomized, multicenter study is clear.

## Introduction

The appearance of pulmonary metastases in patients treated for urogenital cancers represents an undesirable complication. The symptomatology is almost inexistent, so the diagnosis is made by using a radioimagistic method, via radiography and CT-scan. For this reason, a meticulous tracking of the metastases is a requirement both for the patient and the doctor. 

 The malignant disease’s ability to metastasize remains one of the major obstacles when treating patients with cancer. The lungs are one of the most common organs in which cancer metastasizes, and approximately 30% of all cancer patients will develop lung metastases [**1**]. The change from loco-regional to systemic disease usually renders the patient beyond surgical treatment, as local treatment with surgery in a systemic disease is usually considered without benefit. When the systemic disease is present, chemotherapy is the mainstay of treatment and radiation and/or surgery is reserved for palliative measures only. However, numerous retrospective studies have shown that metastasis resection limited to the lung may be associated with prolonged survival. 

 The indication of metastasectomy is currently limited to patients undergoing treatment of the primary tumor (surgical, chemotherapy and possibly radiotherapy). No prospective, randomized studies have been published and most series compare highly selected patients with historical data for patients with no resection for metastases. There is little available information on long-term survival in patients surgically treated for urogenital cancers lung metastases at present.

## Objectives

The purpose of this article is to demonstrate the usefulness of surgery in patients treated for malignant tumors of urogenital organs, which present during the diseases, evolution secondary determinations in the lungs. It also tries to reveal results from other studies published in literature.

## Material, method and search strategy

 This study retrospectively analyses the patients who underwent lung resections for metastases in our department between 2008 and 2013 and compares the results with data published from other centers. 

 Studies and articles for the review were identified by using online searches via www.Pubmed.com, regarding the topic of pulmonary metastasectomy. Several searches were conducted to retrieve all the potentially relevant articles. Terms and keywords associated with pulmonary metastasectomy were used and included, for example, "pulmonary metastases", "pulmonary resection" and "urogenital cancers". None of the retrieved studies were specifically excluded from this review. All the identified studies were retrospective case series. 

## Results

 148 patients with lung metastases were treated in our department between 2008 and 2013, but only 26 had metastases from urogenital cancers. The distribution per years is illustrated in Table 1. 

**Table 1:** The distribution of lung metastases resections per years (n=148)

Year	Urologic cancers (UG)	Genital cancers (GC)	Breast cancers (BC)	Other cancers
2008	0	2	1	7
2009	0	2	3	23
2010	1	1	1	18
2011	2	4	3	18
2012	3	7	2	23
2013	2	2	3	20
total	8	18	13	109

**Fig. 1:**
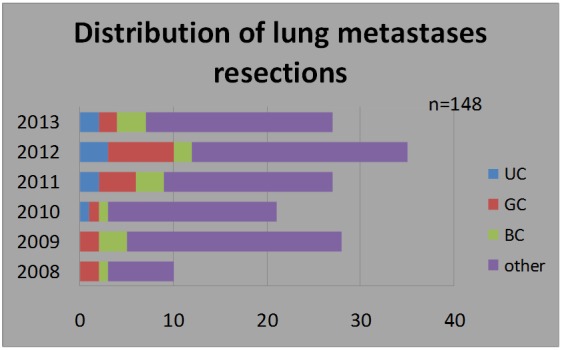
Distribution of lung resections for metastases per year in our clinic (n=148)

**Fig. 2:**
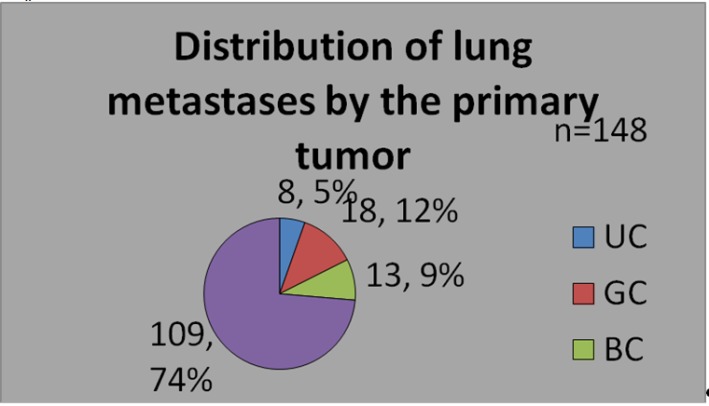
Distribution of each type of metastase (n=148)

 Among these patients, 8 (5.41%) had lung metastases after urologic cancers (UC), 18 (12.16%) were operated for second determinations after genital cancers (GC), 13 (8.78%) after breast tumors metastases and 109 (73.65%) had lung metastases from other type of tumors (**Fig. 2**). 

 The 1-month survival was 100%, but the 6-months survival was 100% (for UC), 94.44% (for GC), 84.62% (for BC) and 87.16%, respectively (for other type of primary tumors). 

 Pulmonary metastasectomy in urogenital cancers occupies the second place among resections for pulmonary second determination in our clinic as it can be seen in Fig. 2. 

**Fig. 3:**
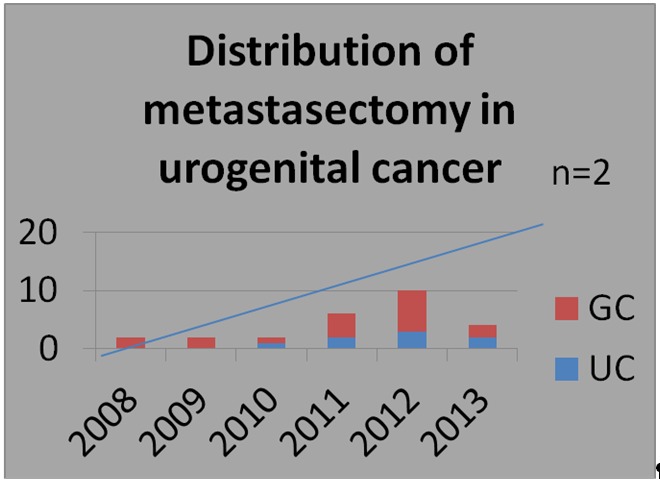
Distribution per years of lung metastasectomy in urogenital cancers (n=26). The trend line shows a slow increase in the number of surgical interventions

 In the last few years, the number of surgical interventions for lung metastases in urogenital cancers has increased (**Fig. 3**). These interventions are usually requested by oncologists considering a better outcome for patients. 

## Discussions 

Although lung metastasis excision thoracotomy in various types of tumors is well known and documented, the data are not clear and surgery is still questionable considering the metastases of urogenital cancers. 

 A notable study considering the metastasis resections is the one made on 133 patients who underwent pulmonary metastasectomy for uterine malignancies enrolled in the Metastatic Lung Tumor Study Group of Japan between March 1984 and February 2002. The 5- and 10-years survivals after the surgical resection in all cases were 54.6% and 44.9%, respectively. The 5-years survivals for each histological type were estimated to be 46.8% for squamous cell carcinoma (n = 58), 40.3% for cervical adenocarcinoma (n = 13), 75.7% for endometrial adenocarcinoma (n = 23), 86.5% for choriocarcinoma (n = 16), and 37.9% for leiomyosarcoma (n = 11). In conclusion, pulmonary metastasectomy for uterine malignancies is a safe and acceptable treatment to improve survival. Patients with a disease-free interval (DFI) of 12 months or more are good candidates for this treatment if there is adequate control of the primary tumor without extra pulmonary metastasis. On the other hand, a poor survival was observed in those with a primary tumor in the cervix, at least 3 metastases and a tumor larger than 3 cm [**2**]. 

 Similar results were obtained in a retrospective study in USA, made on 103 patients (treated for tumors of the uterine corpus - 37 patients, endometrium - 23, cervix - 7, ovaries - 2, and vagina - 1) which states that the factors that negatively influence the surviving are: primary cervix tumor (p < 0.001) and DFI between gynecologic surgery and pulmonary resections < 24 months (p < 0,004) [**3**]. The difference between the DFI statistic values of these two studies is the origin of the various primary tumors. 

 On a smaller scale but not devoid of importance is the study made in Madrid, Spain, on 27 patients, which demonstrates a significant survival prolongation in patients with metastasis resections of the lung [**4**]. A postoperative evolution was influenced by a histological type of the primary tumor and DFI. The survival rate at five years from the diagnosis and excision of the metastasis was the following: 

 - Endometrial carcinoma – 100%; 

 - Cervix cancer – 62,5%; 

 - Uterine corpus sarcoma – 60%. 

 Regarding the evolution and prognosis of uterine sarcomas, a study conducted in New York on a group of patients treated for pulmonary metastases should be noted. The inclusion criteria were prior hysterectomy for uterine sarcoma, no extra thoracic tumor, known disease thought to be resectable, histology consistent with uterine sarcoma and no medical contraindication to thoracotomy. 75% had unilateral lesions, 51% had one lesion, and 70% had nodules greater than 2 cm. The 5- and 10-years survival after hysterectomy for uterine sarcoma was 65% and 50%, respectively, with a mean follow-up of 89 months. The five- and ten-years survival after the resection of pulmonary metastases was 43% and 35%, respectively, with a mean follow-up of 25 months. Unilateral vs. bilateral disease was a significant predictor of survival after pulmonary resection (p = 0.02). Metastases size and number, DFI and patient age were not significant [5,6]. 

 On the other hand, there is the study of a group of doctors from Greece regarding the natural history of 100 patients with pulmonary metastases (PM) from uterine cancer. Lung metastases were found at the time of the diagnosis of the primary tumor in 22 patients. In the remaining 78 patients with PM appearing after the primary therapy, the mean interval time between primary diagnosis and PM was 29.4 months, whereas between PM and death was 15.7 months. What is important to be noted is that of all the patients with lung metastases, 75% did not survive the 1st year; however, 6% survived more than 5 years after the diagnosis of metastatic disease. Patients with isolated PM presented a prolonged survival (36.1 months, p=0.001), whether treated medically or with pulmonary resection. 

 The results of a retrospective study on 243 patients admitted over a five-year period with a diagnosis of carcinoma of the cervix reviewed to determine the frequency of pulmonary metastasis, the relationship between the stage of the primary lesion and the incidence of pulmonary metastasis, and the relationship between the DFI and the incidence of pulmonary metastasis are presented in Table 2 [**7**].

**Table 2:** Carcinoma of the cervix (retrospective observations, n=243)

Carcinoma of the cervix	Pulmonary metastases (PM)	Disease-free interval (DFI) - months
Stage I	4,24%	39
Stage II	13%	37,3
Stage III	7,4%	18
Stage IV	57%	< 1

 Considering the renal cell carcinoma, approximately 25–30% of the patients will have a metastatic disease at diagnosis and a further 30% of the patients will eventually develop metastases, the lungs being the most common target organ [**8**]. Previous studies have demonstrated 5-years survival rates between 31% and 53% from renal cell carcinoma, after pulmonary metastasectomy [**9-11**]. Of the studied prognostic factors, the completeness of resection was found to be a significant prognosticator of survival in all the studies. The number and size of metastases also influenced survival, with few and small metastases having a positive effect on postoperative survival. Further, with a longer DFI, a better survival was reported compared with short DFI.

 Although in our clinic it represented 20%, the surgical pathology of lung metastases of urogenital tumors represents less than 2% of all cases with secondary pulmonary determinations, out of which the most common are renal cell carcinoma and endometrial carcinoma. For future studies concerning this subject, one must take into consideration the analysis of the cases to be handled:.

 - Origin of the primary tumor;.

 - Histopathologic type;.

 - Tumor grading;.

 - DFI;.

 - Number and size of metastases;.

 - Unilateral vs. bilateral disease;.

 - Resection within oncological margins..

**Fig. 4:**
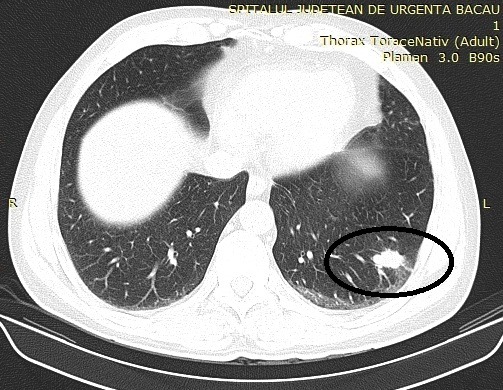
Left pulmonary metastasis on a 51yo patient treated for endometrial carcinoma

**Fig. 5:**
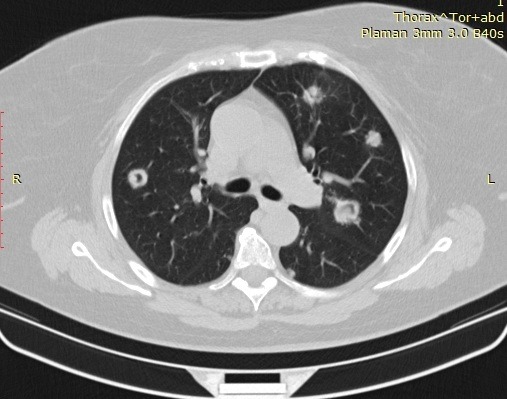
Multiple lung metastases on a 72yo patient treated for cervix tumor

## Conclusion

The appearance of pulmonary metastases is a common and asymptomatic complication of genitourinary cancers and it should be monitored periodically. Resections for lung metastases of high selected patients with urogenital cancer present minimal risks and can prolong survival. So, the criteria for surgery which proved to have positive predictive value that should be taken into account are the following: prolonged DFI, unilateral metastases, the absence of systemic pathologies associated with oncological margins resecability and less than 3 radioimagistic detectable metastases. Further, prognostic factors that determine which patients will benefit from surgery are still not clearly established. In order to determine the way of selecting surgical candidates for pulmonary metastasectomy, further analysis of prognostic factors is evident and the need for a prospective, randomized, multicenter study is clear.
